# Development and Evaluation of Phytosomes Containing *Callistemon citrinus* Leaf Extract: A Preclinical Approach for the Treatment of Obesity in a Rodent Model

**DOI:** 10.3390/pharmaceutics15092178

**Published:** 2023-08-22

**Authors:** Luis Gerardo Ortega-Pérez, Luis Alberto Ayala-Ruiz, Oliver Rafid Magaña-Rodríguez, Jonathan Saúl Piñón-Simental, Asdrubal Aguilera-Méndez, Daniel Godínez-Hernández, Patricia Rios-Chavez

**Affiliations:** 1 Facultad de Biología, Universidad Michoacana de San Nicolás de Hidalgo, Morelia 58000, Michoacán, Mexico; gerardo.ortega@umich.mx (L.G.O.-P.); 1232816g@umich.mx (L.A.A.-R.); oliver.rodriguez@umich.mx (O.R.M.-R.); 1434491c@umich.mx (J.S.P.-S.); 2 Instituto de Investigaciones Químico Biológicas, Universidad Michoacana de San Nicolás de Hidalgo, Morelia 58000, Michoacán, Mexico; amendez@umich.mx (A.A.-M.); dgodinez@umich.mx (D.G.-H.)

**Keywords:** antioxidant capacity, bioavailability, anti-obesogenic, phosphatidylcholine

## Abstract

*Callistemon citrinus* has several biological effects; it is anti-inflammatory, anti-obesogenic, antioxidant, hepatoprotection, and chemoprotective. Its bioactive compounds include terpenoids, phenolic acids, and flavonoids which have low oral bioavailability and absorption. This study aimed at developing phytosomes of *C. citrinus* to improve oral bioavailability and absorption. Phytosomes were formulated with soybean phosphatidylcholine and *C. citrinus* leaf extract using the thin layer sonication method. Phytosomes were evaluated by scanning electron microscopy (SEM), entrapment efficiency, solubility, and particle size determination. Antioxidant capacity and total phenolic, flavonoid, and terpenoid contents were also measured. The in vivo anti-obesogenic activity was evaluated. Phytosomes loaded with *C. citrinus* (P *C.c*) extract had small spherical shapes. The average particle size was 129.98 ± 18.30 nm, encapsulation efficiency 80.49 ± 0.07%, and solubility 90.00%; the stability study presented no significant changes in the average particle size at 20 °C. P *C.c* presented high antioxidant capacity. For the first time, ellagic acid is reported in this plant. The in vivo obesity study showed a strong anti-obesogenic activity of phytosomes with *C. citrinus* to reduce 40% body weight as well as morphometric and biochemical parameters.

## 1. Introduction

Phytosomes are a delivery system of drug or plant extracts prepared with phospholipids using different types of solvents [1]. Compared with drug or plant extracts, phytosomes have a better stability profile, avoid destruction of the phytoconstituent by digestive enzymes and microbiota, increase permeability through membranes, increase bioavailability, and improve compound efficiency [2].

Oxidative stress is not only a common feature of obesity, cardiovascular, neurological, and autoimmune diseases but can also be found in aging [3]. During oxidative stress, there is an increase in reactive oxygen and nitrogen species (ROS/RNS) that are produced by endogenous and exogenous sources [4].

Antioxidants can inhibit, decrease, delay, or directly scavenge free radicals and neutralize oxidants. They act as reducing agents and metal chelators, which convert hydroperoxides into stable compounds. Transferrin, metallothionein, and ceruloplasmin are specific metal-binding proteins considered antioxidant agents and their mechanisms include binding pro-oxidant metal ions, such as iron and copper [5]. The intake of antioxidants may contribute to protecting against the damage produced by reactive oxygen species [6]. The antioxidant activity in plants is due to the phenolic, flavonoid, and terpene compounds found in them [7].

The major compounds found in plants and food are polyphenols and flavonoids. Despite that they can be found in high concentrations, they also need to be available for absorption during gastrointestinal to have beneficial effects. However, these compounds often have low bioaccessibility and bioavailability, which could be due to a number of factors that affect their absorption, stability of the compounds, and the acidic pH of the stomach and microbiota [8,9]. Gastrointestinal pH has an important role in the absorption and bioavailability of oral drugs. In the fasting state, the normal stomach pH is approximately 2.18 ± 0.18 [10]. A change in pH has an impact on the dissolution, solubility release, and stability of drugs [11]. Quin et al. [12] found that the total polyphenol and flavonoid contents of green tea infusion, at pH 1.2, decreased to 65% and 60%, respectively. In addition, the antioxidant activity was reduced as well, leading to low bioaccessibility. Polyphenols are transformed into oligomeric phenols by acidic pH in the stomach. Terpenes containing polar groups at low gastric pH allow bioaccessibility [13]. In summary, pH changes have effects on bioaccessibility that have a strong connection with bioavailability.

*Callistemon citrinus* (Myrtaceae) has been reported to have many biological effects, including antimicrobial, anti-inflammatory, antioxidant, hepatoprotective, and anticarcinogenic [14,15]. Recently, Ortega-Pérez et al. [16] reported that *C. citrinus* leaf extract has anti-obesogenic activity and reduces the oxidative stress observed in obesity. *C. citrinus* has many terpene compounds such as 1-8-cineole, limonene, and α-terpineol [17]. Ayala-Ruiz et al. [18] showed that the main role of these terpenes is to reduce oxidative stress generated by obesity in the animal model. The leaves and stems of *C. citrinus* presented phenolic and flavonoid compounds, such as eucalyptine, blumenol, gallic acid, and protocatechuic acid [19].

Despite the great biological activities of *Callistemon citrinus*, there are few studies about the application of nanoparticles with this plant. The biosynthesis of silver oxide nanoparticles from the aqueous leaf extract of *Callistemon lanceolatus* (*C. citrinus*) proved the in vitro antioxidant capacity and brine shrimp lethality [20]. Paosen et al. [21] reported the synthesis of silver nanoparticles from the Myrtaceae family and the characterization of their antibacterial activity. Silver nanoparticles from leaves, flowers, and seeds of *C. citrinus* exhibited antiplasmodial and antibacterial activity without toxicity [22]. Gold nanoparticle from the seed of *C. citrinus* has antibacterial activity but no antitrypanosomal activity, unlike the extract obtained by the same seed which exhibits both properties [23]. Poly (lactic-co-glycolic acid) nanoparticles loaded with *C. citrinus* phenolics showed anticancer activity against three breast cancer cell lines with 69% growth inhibition [24]. Recently, the use of *C. citrinus* silver nanoparticles from leaf aqueous extract was tested for antibacterial activity [25]. Nanotechnology is a delivery system that can be classified into two groups: inorganic as gold, silver, and copper, and organic as liposomes and polymeric nanoparticles [26]. When novel drug delivery technology is used, instead of traditional drug delivery, side effects are reduced whereas safety and efficacy are improved [27].

Obesity is a global health problem. In the Pacific Island states, 50% of the population is obese. In the United States, one-third of adults are obese [28]. In 2030, more than one billion adults and 50 million children and adolescents will be considered obese [29]. The treatment of obesity is not limited to lifestyle modifications and diets. The most common drugs used to control obesity are orlistat (pancreatic lipase inhibitor), phentermine (sympathomimetic amine), liraglutide (glucagon-like peptide 1 receptor agonist), and naltrexone-bupropion (opioid antagonist and a dopamine and noradrenaline reuptake inhibitor). However, all of them have undesired side effects [30] that can be reduced using natural products from plants as a strategy against obesity [31].

This study aimed at encapsulating *Callistemon citrinus* leaf extract in a phosphatidylcholine complex to enhance its bioavailability and absorption and prevent weight gain. This paper demonstrates that the phytosomes of *Callistemon citrinus* extract had a small size, high entrapment efficiency, and good solubility and stability. The anti-obesogenic activity was also evaluated using male Wistar rats fed with a hypercaloric diet.

## 2. Materials and Methods

### 2.1. Preparation of Callistemon citrinus Leaf Extract

Four-year-old leaves of *Callistemon citrinus* (Curtis) Skeels (Myrtaceae) plants were collected in the city of Morelia, Michoacán, Mexico. The plant voucher specimen EBUM23538 was identified by Professor Patricia Silva at the Biology School of Universidad Michoacana de San Nicolas de Hidalgo. The fresh leaves were macerated in a 1:10 ratio (*w*/*v* 96% ethanol) at room temperature for 5 days. Then, the extract was concentrated by a rotary evaporator at 45 °C. The yield was 20%. The extract of *Callistemon citrinus* was prepared according to the methodology reported by Lopez-Mejia et al. [32]. The authors concluded that the extract should be prepared with leaves of different four-year-old plants to ensure the highest concentration of its major compounds, as well as high antioxidant capacity.

### 2.2. Phytosome Preparation

To prepare phytosomal complex, the same concentration (200 mg/b.w.) of *Callistemon citrinus* and phospholipids were used. This dose has therapeutic efficacy against the inhibition of oxidative stress [15,32] and obesity amelioration [16,18].

Phytosomes were prepared using the assays reported by Baradan et al. [33] and Álvarez-Cortes [34], with slight modifications. The mixture contained 50 mL of hydration media (0.01 M phosphate buffer solution, 150 mM NaCl, pH 7.4), 1.25 g of *Callistemon citrinus* extract, 1.25 g of soybean phospholipids, and 0.72 g of Tween 80; 1% of ethyl acetate was added to improve solubility in the solution. The emulsion was formed using a VCX 500 ultrasonicator with an amplitude of 25% for 10 min at 10 °C. Phytosomes had a stoichiometric ratio of 1:1.

The phytosome complex was placed in an amber-colored glass bottle and stored at room temperature. Design Expert 11.0.5, an experimental design with response surface methodology of central composite design, was used to prepare phytosomes. Lecithin concentration (%*w*/*v*) and rotation speed (rpm) were selected as independent variables. Then, the effect of these variables on the vesicular size and entrapment efficiency of the phytosomes were assessed. All procedures were protected from light. Finally, to corroborate the preparation, the phytosomes were observed under optical microscopy.

#### 2.2.1. Lyophilization and Scanning Electron Microscopy (SEM)

Phytosome samples were frozen at −80 °C overnight; afterward, lyophilized in a high vacuum of 34 Pa using a lyophilizer (Labconco Plus 12; Labconco, Kansas City, MO, USA) for 8 h with a condenser at −43 °C. Lyophilized phytosomes were stored in a sealed glass ampoule at 4 °C. One drop of lyophilized sample was placed on a brass electron microscope tube and coated with copper particles for sputtering. Representative images of the samples were taken and particle diameters were calculated using scanning electron microscopy (JEOL JSM-7600F SEM) with a voltage of 20.0 KV at a working distance of 15.1 mm. Details of the morphological structure of the phytosomes were observed at up to an amplitude of 10,000× and a working distance that allowed minute observations with increasing depth of focus.

#### 2.2.2. Particle Size

Particle size was measured with a Nano Particle Analyzer SZ-100, based on the principle of dynamic light scattering; Ludox TM silica was used as reference material [35]. Ludox TM-50 was diluted to 10% using 0.01 M KCL. A total of 10 mL of KCl/LUDOX solution was filtered through a 2.5 µm filter. The samples were placed in a plastic cuvette and analyzed at a 90° scattering angle. All the batches were analyzed in a triplicate manner and mean and SD were calculated. Table 1 shows the measurement conditions to determine the particle size.

#### 2.2.3. Stability Study

The stability analysis was assessed by storing the phytosomes at 20 ± 2 °C and 4 ± 1 °C and the particle size was measured 1, 3, 5, and 10 days after storing. Later, it was measured after three and a half months.

#### 2.2.4. Study of Vesicular Entrapment/Encapsulation and Solubility

The entrapment efficiency of *C. citrinus* phytosomes was measured using UV-visible spectrophotometer [36]. A total of 1 mL of dialyzed vesicular suspension was taken and diluted with 0.1 mL of Triton X-100. The solution was centrifuged at 1350× *g* for 5 min and the supernatant was diluted with ethanol. The amount of drug entrapped was analyzed spectrophotometrically at a maximum of 425 nm against ethanol containing Triton X-100 as blank. Equation (1) computes the efficiency of entrapment (EE); Tdrug is the total amount of drug; Edrug is the extract entrapment in the formulation (phytosome); and Udrug is the extract not entrapped in phytosomal formulation.

(1) $$\mathrm{EE}=\frac{\mathrm{Edrug}}{\mathrm{Tdrug}}\times 100\%=\frac{\mathrm{Edrug}}{\mathrm{Edrug}+\mathrm{Udrug}}\times 100\%=\left(1-\frac{\mathrm{Udrug}}{\mathrm{Edrug}\;+\;\mathrm{Udrug}}\right)\times 100\%$$

Solubility analysis was calculated by dissolving 2 mg of each of the complexes formed (soybean phospholipid particles) and *C. citrinus* leaf extract in 5 mL of different solvents in small volumetric flasks. The solutions were stirred continuously for 1 h [37]. The experiments were performed in triplicate.

### 2.3. In Vitro Antioxidant Activity

#### 2.3.1. DPPH Radical Assay

The 1,1-diphenyl-2-picrylhydrazyl (DPPH) assay was performed as reported by Kamarac et al. [38]. The solution reaction contained 10 μL of the sample (*C. citrinus* leaf extract or phytosome at 200 mg), 90 μL of methanol, and 2 mL of methanolic solution of DPPH 0.1 mM, which were mixed and incubated in the dark for 60 min at room temperature; its absorbance was measured at 517 nm. Trolox (25–800 µM) was used as standard.

#### 2.3.2. ABTS Radical Scavenging Assay

The 2,2′-Azino-bis (3-ethylbenzothiazoline-6-sulfonic acid) (ABTS) assay was performed as reported by Rufino et al. [39], with slight modifications. A total of 2.6 mM potassium persulfate solution was mixed in equimolar amounts with ABTS (ready to use, Sigma); then, the solution was stirred in the dark for 3 h at 27 °C. This working solution was diluted with ethanol to obtain an absorbance from 0.8–0.9 at 734 nm. For the tests, 1 µL of *C. citrinus* leaf extract (200 mg) and 1 µL of the phytosomes (200 mg) were used, and 49 µL of absolute ethanol and 950 µL of working solution were added. Subsequently, the absorbance at 734 nm was determined after 6 min of starting the reaction.

#### 2.3.3. Ferric-Reducing Antioxidant Power (FRAP) Assay

The FRAP assay was performed as reported by Thaipong et al. [40]. Working solution contained 10 mM 2,4,6-tri [2-pyridyl-s-triazine] (TPTZ) in 40 mM HCL, 20 mM ferric chloride (FeCl_3_.6 H_2_O), and 300 mM sodium acetate buffer (pH 3.6) in a 1:1:10 ratio. A total of 0.1 mL of sample was mixed with 1.5 mL working solution and allowed to stand at room temperature for 20 min in darkness. Then, the absorbance was measured at 593 nm. Results were expressed as mean values ± one standard deviations. Trolox standards ranged from 25 to 800 µM.

#### 2.3.4. Determination of Total Phenolic Content

The total phenolic content was determined using the reported by Pripdeevech et al. [41], with slight modifications; in brief, 0.2 mL of the sample and 1.0 mL of Folin–Ciocalteu reagent (1:9 *v*/*v*) were shaken vigorously for 5 min. Then, 1.0 mL of 7% Na_2_CO_3_ and 5.0 mL of distilled water were added. The reaction mixture was allowed to stand for 60 min at room temperature in darkness and its absorbance was measured at 765 nm. Gallic acid was used as standard (0.01–0.4 mM). Total phenolic content was expressed as mg gallic acid equivalent (mg GAE).

#### 2.3.5. Total Flavonoid Content

The total flavonoid content was determined using the assay reported by Chang et al. [42]. In brief, 0.5 mL of the sample mixed with 1.5 mL of 95% methanol, 0.1 mL of 10% aluminum chloride, 0.1 mL of 1 M potassium acetate, and 2.8 mL of distilled water, stood for 30 min at room temperature in darkness, and the absorbance was measured at 415 nm. Water was used instead of aluminum chloride as blank. Rutin acid was used to calculate the standard curve (0.025–0.5 mg/mL).

#### 2.3.6. Total Terpenoid Content

The total terpenoid content was determined using the methodology described by Chang and Lin [43]. A mixture containing 100 μL of sample (10 mg/mL), 150 μL of vanillin/glacial acetic acid (5% *w*/*v*), and 20 μL of sulfuric acid was incubated at 60 °C for 45 min. The mixture was left on ice for 7 min to stop reaction. Finally, 2.25 mL of glacial acetic acid was added and its absorbance was measured at 548 nm. A total of 1,8-Cineole at 1–6 mg/mL was used as standard.

### 2.4. GC-MS Determination

The samples were analyzed in an Agilent 7890A gas chromatography equipment (Agilent Technologies, Folsom, CA, USA) with an HP5MS30M column (5% phenyl polysilphenylene- siloxane, 30 × 0.25 × 0.25; Agilent Technologies, USA) coupled to an electronic impact ionization quadrupole mass analyzer mass spectrometer. Hewlett Packard 5975C (Hewlett Packard, Palo Alto, CA, USA, EEA). The initial temperature of the oven was 60 °C for 1 min and was increased to 280 °C at 8 °C/min. The injector temperature was 230 °C, the ionization source 230 °C, and the quadrupole temperature 150 °C. Helium was used as carrier gas at a constant flow of 1 mL/min. The mass spectrometer was operated in the EI mode at 70 eV using a range of *m*/*z* 50–500 and the voltage was −1737 V. Total ion chromatograms (TIC) were processed using the automated data processing Software MassHunter Workstation version B.06.00 (Agilent Technologies, Inc.). To identify the different compounds, the mass spectrum of each compound detected was compared to those in mass spectral databases (Wiley 275 and US National Institute of Science and Technology (NIST) V. 2.0. The quantities of compounds were calculated from a standard calibration curve using 1,8-cineole at range 1–0.2 mg/mL.

### 2.5. HPLC Determination

Phenolic acids were quantified by using a high-performance liquid chromatograph (HPLC, Agilent 1260 Infinity Series), equipped with a quaternary pump, auto sampler, column oven, diode array detector (DAD), and Express 90 analytical column. Å C18, 250 × 4.6, 5 µm. The column temperature was 40 °C with an injection volume of 5 µL, the flow was 0.7 mL/min. The mobile phases were A: methanol and B: 1% formic acid. The gradient elution was: 0–5 min: 2% A; 5–15 min: 2–15% A; 15–30 min: 15–25% A; 30–35 min: 25–35% A; 35–45 min: 35–55% A; 40–50 min: 55% A; 50–55 min: 55–2% A; 55–60 min: 2% A; Post time: 5 min. The DAD detector: 255, 270, 280, 310, 322, 355, 370 nm. Eleven available HPLC grade phenolic markers were considered (gallic acid, 4-hydroxybenzoic acid, chlorogenic acid, caffeic acid, vinylic acid, syringic acid, p-coumaric acid, ferulic acid, synaptic acid, ellagic acid, t-cinnamic acid, quercetin, and rutin).

### 2.6. Anti-Obesity Evaluation of Phytosomes

#### 2.6.1. In Vivo Study

##### Animals

Two-month-old male Wistar rats (180–200 g) were obtained from the laboratory animals of the Chemical-Biological Research Institute of UMSNH. All the animals were housed in plastic cages in the following conditions: 12 h light–dark cycle, relative humidity of 60–70%, and a temperature of (23–24 °C). They had ad libitum access to food and water. The animals were kept in the bioterium of the Chemical-Biological Research Institute of UMSNH. All protocols were approved and conducted in accordance with the guide for the care and use of laboratory animals by the Mexican Official Standard (NOM-062-ZOO-1999) and the Ethics Committee of the Universidad Michoacán de San Nicolás de Hidalgo.

#### 2.6.2. Obesity Induction

A high-fat diet (HFD) containing 45.4% normal chow (Rodent diet brand Purina rat chow), 14.8% lard, 14.8% vegetable fat, and 25% fructose was daily prepared as reported in [16]. Fifty-four male Wistar rats were randomly divided into 9 (*n* = 6) groups to be fed. Group 1 (chow diet), Group 2 (chow diet plus vehicle), Group 3 (chow diet plus *C. citrinus* extract 200 mg/kg), Group 4 (HFD), Group 5 (HFD plus *C. citrinus* extract 200 mg/kg), Group 6 (HFD plus phytosomes loaded with *C. citrinus* (P *C.c*) 50 mg/kg), Group 7 (HFD plus P *C.c* 100 mg/kg), Group 8 (HFD plus P *C.c* 200 mg/kg) and Group 9 (HFD plus orlistat 5 mg/kg). Treatments were administered by oral gavage once daily at 9.00 a.m. in the home cage for 15 weeks. The animal’s age at the end of the treatment was 23 weeks. All blood samples were collected after 12–13 h of fasting by cardiac puncture. After blood collection, the animals were anesthetized with pentobarbital sodium injection (150 mg/kg), and all tissues were taken, washed, and stored at −80 °C for subsequent analysis.

#### 2.6.3. Measurement of Morphometric and Biochemical Parameters

Rats were weighed weekly. The percentage of weight gain, adiposity index, and Lee index were calculated as reported by Ortega-Pérez et al. [16]. Plasma glucose, triacylglycerol, and cholesterol were measured using enzymatic colorimetric kits SPINREACT^®^ following the manufacturer’s protocols.

### 2.7. Statistical Analysis

One-way ANOVA is a parametric method that can be used to determine if two or more groups of data are statistically different. Parametric tests make assumptions about the population distribution of the sample and in nonparametric tests the distribution of a population is unknown. This study selected a parametric test because it is more likely to detect significant differences with these methodologies than the use of nonparametric methods. The test results were expressed as mean ± standard error (SEM) or standard deviation (SD). Data were analyzed using GraphPad Prism (version 8.0) by one-way analysis of variance (ANOVA). To determine statistical differences (a, b, c) of nano-phytosomes, and morphometric and biochemical parameters between groups, Tukey’s multiple comparison test was conducted. * *p* ≤ 0.05 is a statistically significant result. Tukey’s honestly significant difference (HSD) test, is a post hoc test used in ANOVA to compare all possible pairs of means. When conducting ANOVA and finding a significant difference among group means, a post hoc test like Tukey’s is needed to determine whether the specific group means significantly differed from each other.

## 3. Results and Discussion

### 3.1. Morphology and Particle-Size Analysis

**Figure 1:**
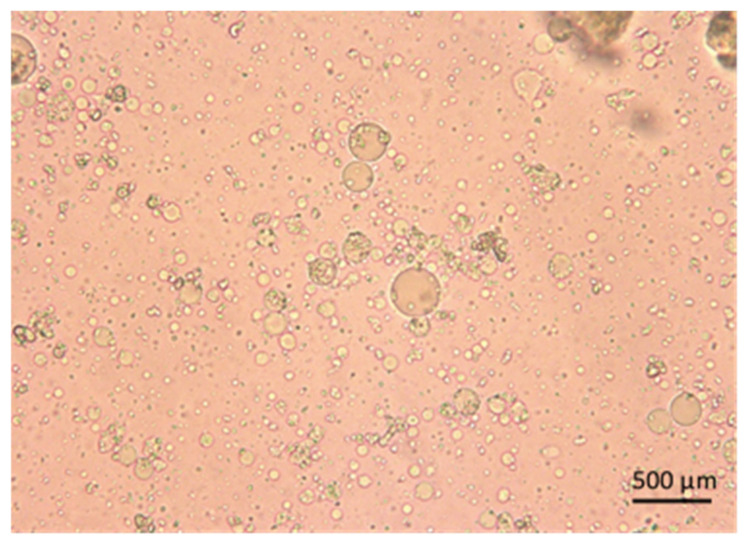
Optical microscope images at a 40× scale of *Callistemon citrinus* phytosomes.

Phytosomes are a strategy used to improve the solubility and bioavailability of herbal extracts [44]. Particle size and phospholipid composition are important factors to obtain these parameters. This study used soybean phosphatidylcholine because this lipid is a main component of membranes and also provides choline, a substrate of choline acetyltransferase to produce the acetylcholine neurotransmitter. Xie et al. [45] reported that using soybean phosphatidylcholine to prepare curcumin-loaded phytosome presented small particle size, high-surface charge, stability, and drug-loading capacity. Figure 1 shows small spherical shapes of phytosomes loaded with *Callistemon citrinus* under an optical microscope at 40×.

**Figure 2:**
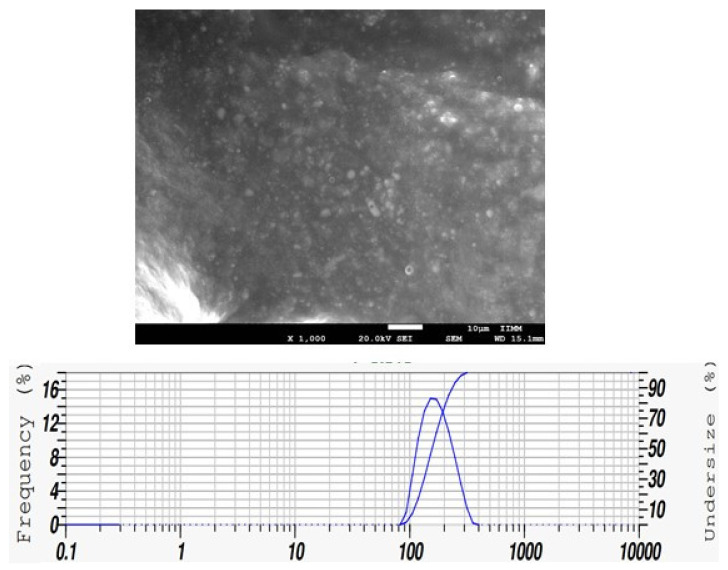
Scanning electron microscope images at scales of 1000×. Particle size was obtained through Nano Particle Analyzer SZ-100 of phytosomes loaded with *Callistemon citrinus* (200 mg/kg).

Scanning electron microscope (SEM) was used to evaluate the size and surface morphology. Figure 2 shows the SEM image confirming that phytosomes have a highly spherical structure. The average particle size of the *Callistemon citrinus* phytosome was 129.98 nm ± 18.30 nm in the emulsion.

### 3.2. Study of Vesicular Entrapment/Encapsulation

The percentage drug entrapment was determined by extracting phytosomes with centrifugation and the supernatant was measured by UV-visible spectroscopy. Table 2 shows the drug entrapment. The results showed that the entrapment efficiency (EE) was about 80.49%. In this way, the encapsulation efficiency of phytosomes is represented by the concentration of unbound *C. citrinus* leaf extract (200 mg/kg); this indicates that the leaf extract and soybean phospholipids react to form the complex with a high degree of entrapment of the leaf extract.

### 3.3. Study of Stability and Solubility

Table 3 shows the stability of the *C. citrinus* phytosome. During the storage period at 20 ± 2 °C, no significant changes in average particle size were observed for the phytosomes. However, low temperatures caused an increase in the particle size up to two folds. Our results indicate that a phytosome loaded with *C. citrinus* remained stable for three and a half months. This result is similar to a phytosome loaded with *Cuscuta reflexa* [46].

The low lipid solubility of some compounds may be the reason for their weak absorption [47]. Thus, the solubility is an important parameter to study. Table 4 shows that *Callistemon citrinus* phytosomes were completely soluble in four solvents and partially soluble in one of them. Assuming 20% for the former and 10% for the latter, *Callistemon citrinus* phytosomes had a 90% of solubility. It follows that *C. citrinus* extract, without and with tween 80, shows 80% of solubility and finally soybean liposomes. The formation of phytosomes with plant extract is based on hydrogen-bonding interaction, which increases the bioavailability and stability of the compounds [48]. Consequently, phytosomes have better lipophilicity and hydrophilicity than bioactive compounds.

### 3.4. In Vitro Antioxidant Activity of Callistemon citrinus Phytosomes

**Figure 3:**
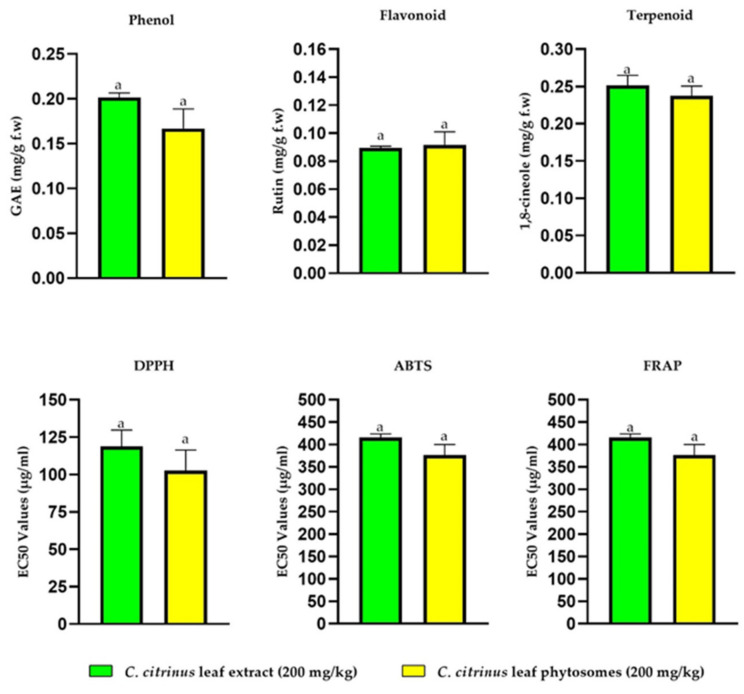
Determination of antioxidant capacity and total phenol, flavonoid, and terpenoid contents of *Callistemon citrinus* leaf extract and phytosomes. Data are expressed with the mean ± standard error (ANOVA followed by Tukey, *n* = 6). The same letter (a) meaning that there is no statistical differences.

DPPH, ABTS, and FRAP are methodologies commonly used to evaluate the antioxidant capacity of plant extracts. DPPH and ABTS are based on the hydrogen or electron-donating capacity and FRAP on the capacity of reducing ferric to ferrous [49]. Ortega-Perez et al. [16] reported the strong antioxidant capacity and the total phenol, flavonoid, and terpene compounds of *Callistemon citrinus* leaf extract. Figure 3 shows that both *C. citrinus* extract and *C. citrinus* phytosomes exhibited significant inhibitory activity against the DPPH and ABTS radicals and a high ability to reduce ferric to ferrous. Many reports have demonstrated the correlation between total phenolic and flavonoid content and their antioxidant activities [50]. This study also found this correlation, suggesting that the compounds produced the antioxidant effect in *C. citrinus*, acting as hydrogen donators, singlet oxygen quenchers, and reducing agents [51].

Figure 3 shows that there are no significant differences between the bioactive compounds and the antioxidant capacity in the *Callistemon citrinus* extract and the phytosomes of *C. citrinus*. This result shows that during the process of creating phytosomes, the bioactive compounds and antioxidant capacity were retained. However, the encapsulation did not significantly improve the activity. This result agrees with Saonere et al. [52], which found antioxidant capacity in a phytophospholipid complex of *Glycerrhiza glabra*.

Phytosomes containing extracts of mulberry and ginger used against the metabolic syndrome improved the antioxidant system and decreased inflammatory cytokines such as IL-6 and TNF-α [53]. The use of phytosome curcumin against paracetamol-induced liver toxicity in mice showed an increase in enzymatic antioxidant activities and the reduction in lipoperoxidation products [54]. Deleanu et al. [55] reported that phytosomes with the extract of ginger rhizomes and rosehips increase the bioavailability, antioxidant, and anti-inflammatory properties in LPS-induced systemic inflammation in mice. These results suggest that the use of phytosomes can improve enzymatic antioxidant properties and reduce inflammation during oxidative stress [33].

### 3.5. Gas Chromatography and Mass Spectrometry Analysis

**Figure 4:**
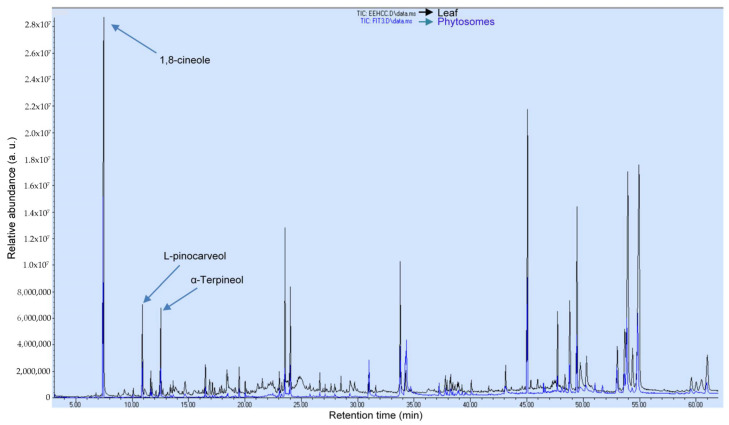
Comparison of terpenoid abundance (arbitrary units) in GC/MS total ion chromatogram of *Callistemon citrinus* leaf (black) and *C. citrinus* phytosomes (blue).

Figure 4 shows the chromatogram of *C. citrinus* extract and *C. citrinus* phytosome analyzed by GC/MS to evaluate the terpenes profile. Terpenes were quantified according to GC/MS. Table 5 shows that 1,8-cineole and α-terpineol were the main compounds of the extract and phytosome. These two monoterpenes have been reported to have hepatoprotective, antiviral, antimicrobial, antioxidant, and anticarcinogenic effects [32,56], suggesting that *C. citrinus* may constitute an alternative pharmacological tool to treat oxidative stress in some diseases.

Table 5 shows the calculated retention indices and a comparison with retention indices found on the NIST home page (www.nistwebbook.com (accessed on 17 March 2023)). A total of 80% of the compounds were identified and only two of them could not be fully identified despite having a Match Factor above 800 (Good Match). According to the NIST library, Match Factor scores > 900 mean an Excellent Match, whereas Match Factors scores in the range of 800–900 are a Good Match. All identified compounds have Factor scores above 800.

### 3.6. High-Performance Liquid Chromatography Analysis

**Figure 5:**
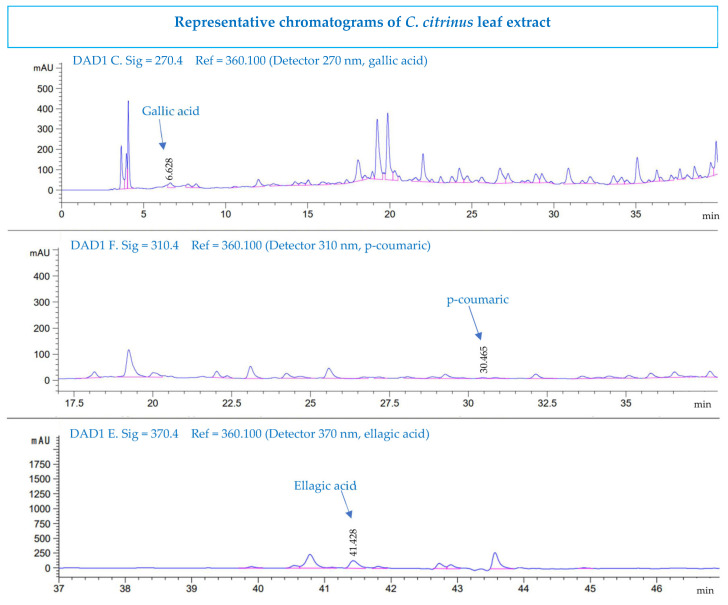
HPLC chromatograms of the *Callistemon citrinus* leaf extract.

**Figure 6:**
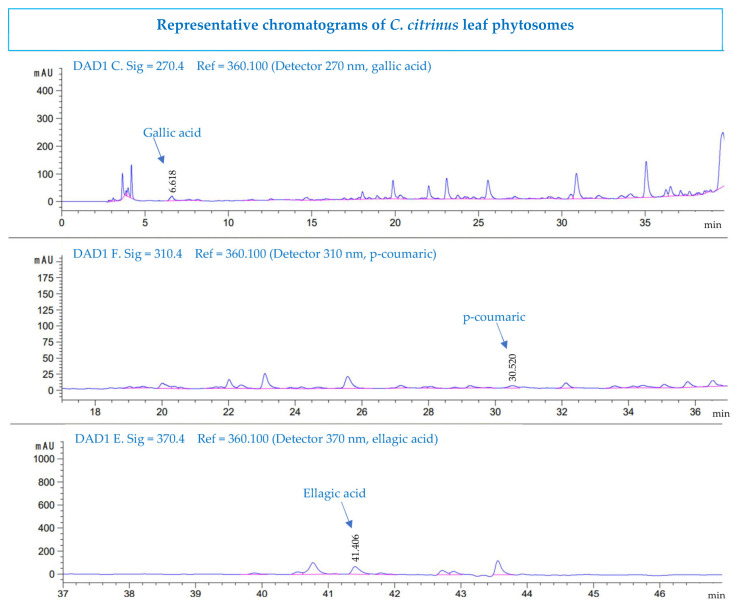
HPLC chromatograms of *Callistemon citrinus* leaf phytosome.

The phenolic and flavonoid compounds were identified according to their retention time in HPLC. Ellagic acid was found for the first time in *C. citrinus*. Figure 5 and Figure 6 show that gallic acid, *p*-coumaric acid, and ellagic acid are the compounds identified in the *Callistemon citrinus* extract and phytosome. These phenolic acids have been reported to have anticancer, antiviral, antioxidant, and anti-inflammatory activities [64]. Table 6 shows that the concentration of gallic acid, p-coumaric acid, and ellagic acid was very similar in the *C. citrinus* extract and in the phytosome.

Until now, silver, gold, and poly (lactic-co-glycolic acid) nanoparticles loaded with *Callistemon citrinus* [22,23,24] have been reported. Nanoparticles have some characteristics that could affect their toxicity as nature, size, mobility, stability, surface aggregation, and storing time [65]. Metal oxide nanoparticles reduced the enzymatic activity of microorganisms [66]. However, the use of phytosomes as a delivery method of natural products has advantages. Phytosomes have an amphiphilic characteristic that allows the extract compounds to interact with the hydrophilic and hydrophobic parts, increasing the therapeutic effect.

### 3.7. Effect of Phytosomes on Morphometric and Biochemical Parameters

**Figure 7:**
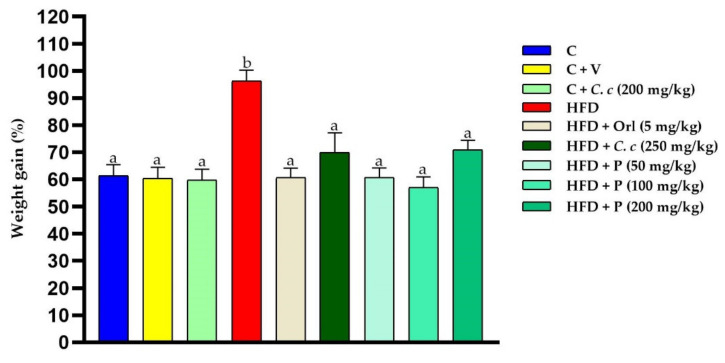
Final body weight percentage in control rats and the different experimental treatments during the 15 weeks. Values are presented as mean ± standard error (ANOVA followed by Tukey, *n* = 6). Statistically different values (a, b) between groups.

The rats fed with HFD showed increased body weight compared to the other groups. Conversely, the administration of phytosomes loaded with *C. citrinus* to animals fed with HFD showed significantly reduced body weight as compared to obese rats (Figure 7). These results agree with Ortega et al. [16]. A previous study showed that *C. citrinus* extract inhibited lipase activity in a dose-dependent manner [16]. Regarding the phytosomal dosage (50, 100, and 200 mg/kg) and *C. citrinus* extract (250 mg/kg), this study showed similar effects in all of them to reduce weight. This study suggests that phytosomes loaded with *C. citrinus* extract have stronger anti-obesogenic activity than *C. citrinus* extract itself; this result is probably due to the high bioavailability, which improves the solubility, allowing to reduce the dose. The Lee and adiposity indices in the HFD group were higher than those in other groups. Also, glucose and triacylglycerol levels increased in the HFD group, contrary to the rest of the groups (Table 7).

*Callistemon citrinus* phytosomal formulation improved oral bioavailability. Even the administration of low doses reduced the morphometrical and biochemical parameters in the treated animals.

## 4. Conclusions

The *Callistemon citrinus* phytosomal formulation improved oral bioavailability, retained the major compounds, and was stable for three and a half months when stored at 20 °C. Phytosomes of *C. citrinus*, even in low doses, reduced morphometrical and biochemical parameters in Wistar rats fed with a high-fat diet. The results also revealed that the supplementation of phytosomes of *Callistemon citrinus* reduced excessive weight in the animals.

## Figures and Tables

**Table 1:** Measurement conditions to determine the particle size.

Temperature	25 °C
Particle	LUDOX (1.45–0.000i)
Dispersion medium	Water
Cell	Plastic
Distribution type	Monodisperse narrow

**Table 2:** Entrapment efficiency of the *Callistemon citrinus* phytosomes.

Parameter	Abs
Tdrug	0.189 ± 0.01
Udrug	0.045 ± 0.07
EE	80.49 ± 0.07%

(Tdrug) is the total amount of drug, (EE) is the efficiency of entrapment, and (Udrug) is the extract not entrapped in phytosomal formulation. The data are expressed with the mean (*n* = 4) and standard deviation (±SD).

**Table 3:** Effect of the temperature on the stability of *Callistemon citrinus* phytosomes at 1, 3, 5, and 10 days and 3.5 months.

Temperature		
Days	20 ± 2 °C	4 ± 1 °C
1	193.62 ± 27.33 ^a^	285.07 ± 14.04 ^ab^
3	218.06 ± 59.55 ^a^	412.80 ± 248.22 ^abc^
5	256.50 ± 29.00 ^a^	454.23 ± 175.28 ^abc^
10	279.64 ± 61.21 ^a^	570.70 ± 132.73 ^bc^
106	283.82 ± 51.87 ^ab^	623.23 ± 142.18 ^c^

Values are the particle size (nm) expressed as mean ± SD (ANOVA followed by Tukey, statistically different values (^a^, ^b^, ^c^) between groups (*p* ≤ 0.05, *n* = 6)).

**Table 4:** Solubility profile of *Callistemon citrinus* leaf extract, *Callistemon citrinus* phytosomes, and soybean phospholipids.

Solvent	*C. citrinus* Extract (200 mg/kg)	*C. citrinus* Extract (200 mg/kg) + Tween 80	*C. citrinus* Phytosomes (200 mg/kg)	Soybean Liposomes + Tween 80	Soybean Liposomes-Tween 80
Distilled water	Partially	Partially	Soluble	Soluble	Micellar shape
Methanol	Soluble	Soluble	Partially	Unsolvable	Soluble
Dichloromethane	Soluble	Partially	Soluble	Soluble	Soluble
Chloroform	Soluble	Soluble	Soluble	Soluble	Partially
Hexane	Partially	Soluble	Soluble	Soluble	Soluble

5 mL of each solvent was added. The solutions were placed under continuous stirring for 1 h; *n* = 4.

**Table 5:** Terpene contents in *Callistemon citrinus* leaf extract and *C. citrinus* phytosomes (GC/MS).

RT	RI_lit_	RI_calc_	Ref. RI_lit_	Match Factor	Prob (%)	Compounds	Extract	Phytosomes
7.47	1041	1059	Silva et al. [57]	972	93.8%	1,8-Cineole	0.613 ± 0.05	0.224 ± 0.04
10.91	1143	1131	Radulovic et al. [58]	950	67.2%	L-Pinocarveol	0.097 ± 0.007	0.030 ± 0.005
11.65	1140	1114	Muselli et al. [59]	883	68.6%	Pinocarvone	0.016 ± 0.003	nd
11.76	1170	1166	Al-Omar [60]	923	63.9%	Borneol	0.0081 ± 0.001	nd
12.54	1172	1143	Boti et al. [61]	952	74.5%	α-Terpineol	0.0894 ± 0.04	0.0233 ± 0.003
23.04	1567	1530	Babushok et al. [62]	929	55.5%	Globulol	0.011 ± 0.002	0.0012 ± 0.001
33.78	2099	2045	Babushok et al. [62]	894	81.2%	Phytol	0.1714 ± 0.03	0.0637 ± 0.01
45.04	2847	2914	Zhao et al. [63]	963	50.4%	Squalene	0.1041 ± 0.01	0.0044 ± 0.001
53.66	-	2886	-	886	59.9%	Unknown 1	0.0957 ± 0.02	0.0364 ± 0.006
54.94	-	2848	-	941	86.5%	Unknow 2	0.8505 ± 0.05	0.2187 ± 0.03

RT retention time (min). RI_lit_ retention index (iu) reported in the literature for 5% phenyl polysilphenylene-siloxane GC column. RI_calc_ retention index obtained through the modulated chromatogram. Ref. RI_lit_ retention index bibliography found in the literature for 5% phenyl polysilphenylene-siloxane GC column. Extract (mg/mL), phytosomes (mg/mL). nd = Not detected. Non-polar retention index (n-alkane scale). The values are the mean ± SD (*n* = 3). Fragment ions (*m*/*z*) of unknown 1: 218 (100), 203, 219, 69, 95, 426 [M+], 411.4. Unknown 2: 189 (100, 95,207,93,135, 426 [M+], 411.4.

**Table 6:** HPLC analysis profile in *Callistemon citrinus* leaf extract and phytosomes.

Compounds	Extract (µg/mL)	Phytosomes (µg/mL)
gallic acid	6.94 ± 0.06	5.93 ± 0.0
4-hydroxybenzoic acid	nd	nd
chlorogenic acid	nd	nd
caffeic acid	nd	nd
Vanillic acid	nd	nd
Syringic acid	nd	nd
*p*-coumaric acid	0.47 ± 0.05	0.65 ± 0.07
ferulic acid	nd	nd
synaptic acid	nd	nd
ellagic acid	74.3 ± 1.3	67.3 ± 1.4
*t*-cinnamic acid	nd	nd
quercetin	nd	nd
rutin	nd	nd

nd = Not detected. Data expressed as mean ± SD (*n* = 3).

**Table 7:** Effect of *C. citrinus* on morphometric parameters, obesity markers, and biochemical determinations in rats.

Measurements	Control	Control + Vehicle	Control + *C. citrinus* Extract (200 mg/kg)	Hypercaloric-fat Diet (HFD)	HFD + Orlistat (5 mg/kg)	HFD + *C. citrinus* Extract (250 mg/kg)	HFD + Phytosomes (50 mg/kg)	HFD + Phytosomes (100 mg/kg)	HFD + Phytosomes (200 mg/kg)
Morphometric parameters									
Abdominal circumference (cm)	20.50 ± 0.45 ^a^	20.50 ± 45 ^a^	21.00 ± 0.45 ^a^	25.50 ± 0.45 ^b^	22.25 ± 0.45 ^a^	20.33 ± 1.36 ^a^	21.0 ± 0.52 ^a^	21.20 ± 20 ^a^	21.50 ± 0.45 ^a^
Nose-to-anus length (cm)	25.25 ± 0.60 ^a^	24.37 ± 0.60 ^a^	24.60 ± 0.54 ^a^	24.41 ± 0.91 ^a^	23.66 ± 0.91 ^a^	24.41 ± 0.91 ^a^	23.80 ± 0.54 ^a^	24.12 ± 0.60 ^a^	23.50 ± 0.60 ^a^
Nose-to-tail length (cm)	46.40 ± 0.42 ^a^	46.40 ± 0.42 ^a^	46.87 ± 0.47 ^a^	45.66 ± 0.38 ^a^	45.71 ± 0.47 ^a^	44.66 ± 1.63 ^a^	45.87 ± 0.47 ^a^	45.75 ± 0.47 ^a^	45.12 ± 0.47 ^a^
Markers of obesity									
BMI (kg/m^2^)	0.67 ± 0.03 ^b^	0.72 ± 0.03 ^ab^	0.70 ± 0.03 ^ab^	0.88 ± 0.04 ^a^	0.72 ± 0.04 ^ab^	0.69 ± 0.09 ^b^	0.66 ± 0.04 ^b^	0.68 ± 0.04 ^b^	0.76 ± 0.04 ^ab^
Adiposity index	2.78 ± 0.55 ^c^	2.77 ± 0.55 ^c^	2.52 ± 0.62 ^c^	9.43 ± 0.62 ^a^	5.56 ± 0.71 ^bc^	6.18 ± 0.39 ^b^	5.98 ± 0.71 ^b^	4.82 ± 0.71 ^bc^	4.02 ± 0.62 ^bc^
Lee index	0.30 ± 0.01	0.30 ± 0.02	0.30 ± 0.01	0.33 ± 0.01 ^a^	0.30 ± 0.01	0.30 ± 0.01	0.29 ± 0.01	0.30 ± 0.01	0.31 ± 0.01
Biochemical parameters									
Triacylglycerol (mg/dL)	90.66 ± 11.64 ^c^	103.66 ± 11.64 ^c^	109.66 ± 11.64 ^c^	202.66 ± 11.64 ^a^	90.66 ± 11.64 ^c^	136.33 ± 66.96 ^b^	103.50 ± 11.64 ^c^	105.33 ± 11.64 ^c^	118.66 ± 11.64 ^b^
Blood glucose (mg/dL)	93.99 ± 8.24 ^a^	101.37 ± 8.24 ^a^	95.59 ± 8.24 ^a^	111.11 ± 8.24 ^b^	100.24 ± 8.24 ^a^	97.00 ± 4.24 ^a^	104.18 ± 8.24 ^a^	92.24 ± 8.24 ^a^	96.65 ± 8.24 ^a^
Total cholesterol (mg/dL)	161.33 ± 2.69 ^a^	160.33 ± 2.69 ^a^	162.00 ± 2.69 ^a^	159.66 ± 2.69 ^a^	156.66 ± 2.69 ^a^	162.00 ± 2.69 ^a^	155.66 ± 2.69 ^a^	161.00 ± 2.69 ^a^	159.30 ± 2.69 ^a^

Values expressed as mean ± SEM (*n* = 6, ANOVA followed by Tukey test, statistically different values (^a^, ^b^, ^c^) between groups; *p* ≤ 0.05).

## Data Availability

Data availability under request.
